# Pre-existing yellow fever immunity impairs and modulates the antibody response to tick-borne encephalitis vaccination

**DOI:** 10.1038/s41541-019-0133-5

**Published:** 2019-09-06

**Authors:** Victoria Bradt, Stefan Malafa, Amrei von Braun, Johanna Jarmer, Georgios Tsouchnikas, Iris Medits, Kerstin Wanke, Urs Karrer, Karin Stiasny, Franz X. Heinz

**Affiliations:** 1 Center for Virology, Medical University of Vienna, Vienna, Austria; 2 Division of Infectious Diseases, Department of Medicine, University Hospital of Zurich, Zurich, Switzerland; 3 Present Address: Department of Medicine, University Hospital of Leipzig, Leipzig, Germany; 4 Present Address: Boehringer Ingelheim RCV GmbH & Co KG, Vienna, Austria; 5 Present Address: Hookipa Pharma, Vienna, Austria; 6 Present Address: Novartis, Rotkreuz, Switzerland; 7 Present Address: Department of Medicine, Cantonal Hospital of Winterthur, Winterthur, Switzerland

**Keywords:** Viral infection, Virology

## Abstract

Flaviviruses have an increasing global impact as arthropod-transmitted human pathogens, exemplified by Zika, dengue, yellow fever (YF), West Nile, Japanese encephalitis, and tick-borne encephalitis (TBE) viruses. Since all flaviviruses are antigenically related, they are prone to phenomena of immunological memory (‘original antigenic sin’), which can modulate immune responses in the course of sequential infections and/or vaccinations. In our study, we analyzed the influence of pre-existing YF vaccine-derived immunity on the antibody response to TBE vaccination. By comparing samples from YF pre-vaccinated and flavivirus–naive individuals, we show that YF immunity not only caused a significant impairment of the neutralizing antibody response to TBE vaccination but also a reduction of the specific TBE virus neutralizing activities (NT/ELISA-titer ratios). Our results point to a possible negative effect of pre-existing cross-reactive immunity on the outcome of flavivirus vaccination that may also pertain to other combinations of sequential flavivirus infections and/or vaccinations.

## Flavivirus vaccines: Yellow Fever vaccine commits ‘original antigenic sin’

Flaviviruses, such as Yellow Fever (YF) virus and Tick-borne encephalitis (TBE) virus, share a certain level of antigenic relatedness, meaning that pre-existing immunity to one flavivirus species can impact the antibody response to a second — a phenomenon known as ‘original antigenic sin’. A team led by Franz X. Heinz at the Medical University of Vienna investigate the impact of pre-vaccination with the YF vaccine (a minimum of one year earlier) on subsequent vaccination against TBE. Overall, YF pre-immunity significantly reduced generation of TBE neutralizing antibodies compared to flavivirus-naive individuals, but there was a striking level of inter-individual variation. Although even the YF-vaccinated individuals produced sufficient TBE neutralizing titers to be considered potentially protective, these findings suggest that the influence of ‘original antigenic sin’ might need to be taken into account for flavivirus vaccination protocols.

## Introduction

Flaviviruses comprise a number of important human pathogens that are transmitted to their vertebrate hosts either by mosquitoes (yellow fever, dengue, Zika, West Nile, and Japanese encephalitis viruses) or by ticks (tick-borne encephalitis and Powassan viruses). Explosive outbreaks and dramatic expansions of endemic regions have been documented in the recent past for Zika, West Nile (WN), and dengue viruses (Den), underlining the impact of flaviviruses as emerging pathogens.^1–4^ All flaviviruses are antigenically related and can induce broadly flavivirus cross-reactive antibodies; cross-protection, however, is not observed among distantly related flaviviruses.^5–7^ In contrast, cross-reactive antibodies may even have a disease-enhancing effect during a subsequent exposure with a different flavivirus.^6,8^ In general, the immunological memory to cross-reactive antigenic sites and the formation of immune-complexes can modulate the antibody response in sequential infections or immunizations with antigenically related viruses or immunogens, respectively. This phenomenon has been referred to as ‘original antigenic sin’ (OAS)^9–11^ and is especially relevant in the context of annual infections and vaccinations with drifting and shifting influenza viruses.^12,13^ Also in the case of flaviviruses, OAS phenomena can play an important role,^14,15^ as a result of sequential exposures to flaviviruses that co-circulate in the same geographical regions, global travel-related exposures, and/or immunization with different flavivirus vaccines, which is the topic of the present work.

Several flavivirus vaccines are licensed and commercially available in one or more countries, and others are in clinical development.^16^ The live YF vaccine (based on the attenuated 17 D strain) is one of the most successful vaccines ever produced and in widespread use in YF-endemic areas of Africa and South America as well as in travelers to these areas.^17^ Two live vaccines are available against Japanese encephalitis (JE) (based on the attenuated SA14-14-2 strain) and a JE-YF chimera,^18^ and recently a tetrameric Den-YF chimeric live vaccine against dengue was licensed in a number of countries.^18^ In addition to these live vaccines, inactivated TBE and JE vaccines are in use, which are both based on purified, formalin-inactivated virions.^18,19^ The induction of antibodies capable of virus neutralization has been described as the major mechanism for conferring long-lasting protection against flavivirus disease and is considered the most important surrogate marker for vaccine-induced protection.^17,20,21^ Neutralizing antibodies bind to the major viral envelope protein (E) and inhibit the viral entry functions (cell attachment and membrane fusion) that are mediated by this protein.^22^

**Fig. 1:**
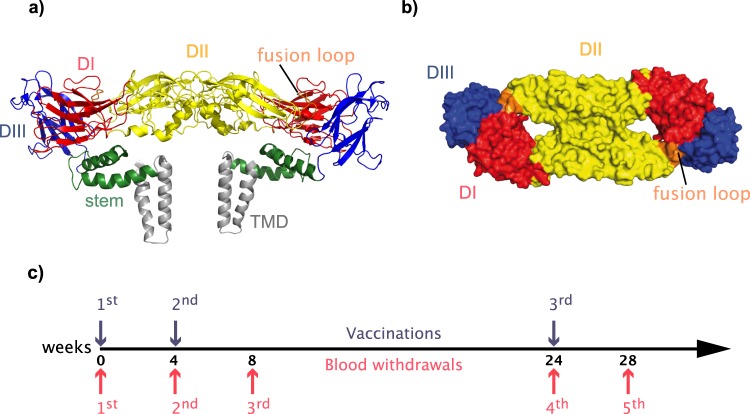
Structure of TBE virus E protein and TBE vaccination schedule. **a** Ribbon diagram of the TBE virus E dimer (PDB: 5O6A;^52^) in side view and **b** surface representation of TBE virus E dimer (PDB: 1SVB;^53^) in top view (lacking the stem and transmembrane domains (TMD)). Color code: DI, red; DII, yellow; DIII, blue; stem, green; TMD, gray; the fusion loop (FL) is highlighted in orange. **c** Time schedule of TBE vaccination (top – blue arrows) and blood withdrawals (bottom – red arrows). The structures were generated with PyMol (Schrödinger, LLC; www.pymol.org) using published PDB files (https://www.rcsb.org)

Mature infectious flavivirus particles contain 180 copies of E that are arranged as 90 homodimers in an icosahedral, herringbone-like arrangement.^6,22,23^ The overall molecular organization of E is very similar for all flaviviruses, with a C-terminal double membrane-spanning anchor, a so-called stem region and an N-terminal external part consisting of ~80% of the amino acids assembled in three distinct domains, termed DI, DII, and DIII (Fig. 1a, b). At the amino acid sequence level, however, the E proteins of different flaviviruses diverge substantially (up to 60%), and cross-neutralization by polyclonal immune sera is usually observed only between relatively closely related viruses that have been grouped into serocomplexes based on this criterion (e.g., TBE and Powassan viruses, WN and JE viruses, or the four dengue viruses are part of the same serocomplexes, respectively).^24^ Broadly flavivirus cross-reactive antibodies are induced to various degrees in the course of flavivirus infections and can be readily observed in serological assays other than neutralization tests (NT), such as ELISA and hemagglutination-inhibition (HI) tests.^6^ The most prominent structural element responsible for the induction of antibodies reacting with all flaviviruses is the fusion loop (FL) at the tip of DII (Fig. 1a, b), which is highly conserved but largely buried in the dimeric structure of E.^6^

The likelihood of individuals being vaccinated on the background of previous flavivirus immunity increases with the availability of flavivirus vaccines in endemic areas as well as their use as travel vaccines. In this context, we investigated whether and in which specific way immunity induced by previous YF vaccination had an influence on the antibody response to TBE vaccination. In addition, our study also focused on the degree of variation of antibody specificities in vaccinated individuals. By comparing the antibody responses in flavivirus-naive and YF pre-vaccinated groups, we demonstrate that pre-existing YF vaccine-derived immunity had an overall negative effect on the neutralizing antibody response to TBE vaccination but resulted in the boosting of cross-reactive, non-neutralizing antibodies. Notwithstanding these overall effects, our analyses also demonstrate strong individual deviations from the average antibody patterns observed in both groups.

## Results

### Antibody response to TBE vaccination

**Fig. 2:**
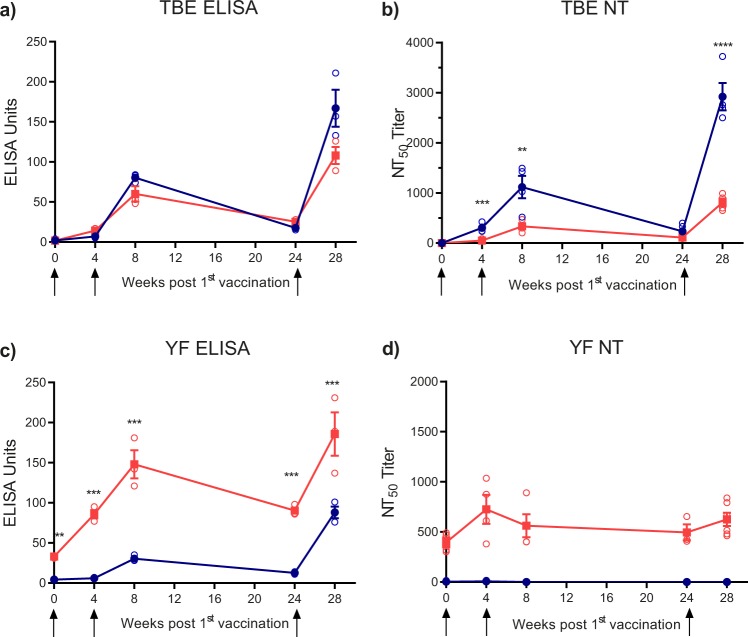
ELISA and neutralization tests of TBE post-vaccination plasma pools. Blue lines and filled symbols (mean values): Plasma pools of flavivirus-naive group. Red lines and filled symbols (mean values): Plasma pools of YF pre-vaccinated group. Open circles show results of independent experiments. Testing was performed in TBE and YF ELISA (**a**, **c**), as well as TBE and YF NT (**b**, **d**) at five different time points in the course of TBE vaccination (Fig. 1c). Arrows indicate time points of vaccination. Error bars represent the standard errors of the means (SEM) calculated from at least three independent experiments. Asterisks indicate significant differences between the two groups at the different time points (measured by *t*-test): **P < 0.01; ***P < 0.001; ****P < 0.0001

Samples were derived from groups of flavivirus-naive and YF-pre-vaccinated individuals who had received TBE vaccination according to a basic vaccination schedule at time points 0, 4, and 24 weeks (Table 1 and Fig. 1c). Blood samples were collected at the time of the first and third vaccination, as well as 4 weeks after each immunization, corresponding to time points 0, 4, 8, 24, and 28 weeks after the first vaccination (Fig. 1c). Pools of the plasma samples from each group at the five time points were analyzed in TBE and YF ELISA using recombinant E as well as in TBE and YF NT and the results are shown in Fig. 2.

**Table 1:** Characteristics of the study cohort

	Flavivirus-naive group	YF pre-vaccinated group
Number	44	28
Sex (female/male)	18/26	11/17
Mean age (range) at study begin	67 (24–86)	69 (27–87)
Average years (range) since YF vaccination		20 (1**–**48)

Plasma samples from TBE-vaccinated individuals

#### TBE response

In the pools of the flavivirus-naive group, TBE ELISA and NT both showed a substantial increase of antibody titers after the 2^nd^ dose, followed by a decline and a further strong boost after the 3^rd^ dose (Fig. 2a, b). Although the YF pre-vaccinated group displayed a similar pattern in TBE ELISA (Fig. 2a), the neutralizing antibody response was substantially lower (Fig. 2b), and this difference was statistically significant for the blood drawings after the 1^st^, 2^nd^ and 3^rd^ vaccination.

#### YF response

In the flavivirus-naive group, relatively few YF ELISA-reactive antibodies were induced, whereas in the YF pre-vaccinated group a strong booster effect was observed (Fig. 2c). This boosting of YF ELISA-reactive antibodies, however, did not significantly change the pre-existing YF neutralizing antibody titers (Fig. 2d), and the plasma pools from the flavivirus-naive group did not contain measurable YF virus-neutralizing antibodies at any time point of the vaccination schedule (Fig. 2d).

**Fig. 3:**
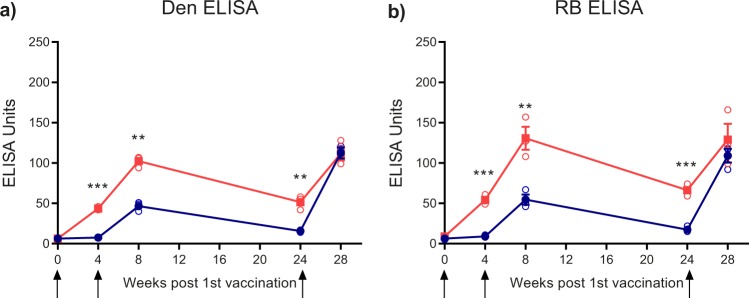
Den and RB ELISA of TBE post-vaccination plasma pools. Blue lines and filled symbols (mean values): Plasma pools of flavivirus-naive group. Red lines and filled symbols (mean values): Plasma pools of YF pre-vaccinated group. Open circles show results of independent experiments. Testing was performed in **a** Den and **b** RB ELISA at five different time points in the course of TBE vaccination (Fig. 1c). Arrows indicate time points of vaccination. Error bars represent the SEM calculated from at least three independent experiments. Asterisks indicate significant differences between the two groups at the different time points (measured by *t*-test): **P < 0.01; ***P < 0.001

To analyze the development of broadly flavivirus cross-reactive antibodies more specifically, we performed ELISAs with the soluble E proteins (sE) of two flaviviruses that are distantly related to both YF and TBE viruses: The mosquito-borne Den 1 virus and the ‘no known vector’ Rio Bravo (RB) virus.^25^ The results obtained from these analyses (Fig. 3a, b) are consistent with the YF virus E-response (Fig. 2c), showing a strong initial boost of broadly flavivirus cross-reactive antibodies in the YF pre-vaccinated group (red lines in Fig. 3a, b). Compared to the reactivities after the 2^nd^ vaccination (time point 8 weeks), no further increase of such antibodies, however, was observed after the 3^rd^ vaccination (time point 28 weeks). The flavivirus-naive group developed significantly lower cross-reactivity after the 1^st^ and 2^nd^ vaccinations (blue lines in Fig. 3a, b). This difference between the two groups, however, disappeared at time point 28 weeks due to a substantial boost after the 3^rd^ vaccination in the flavivirus-naive group (Fig. 3a, b).

### Antibody depletion with Rio Bravo E protein

**Fig. 4:**
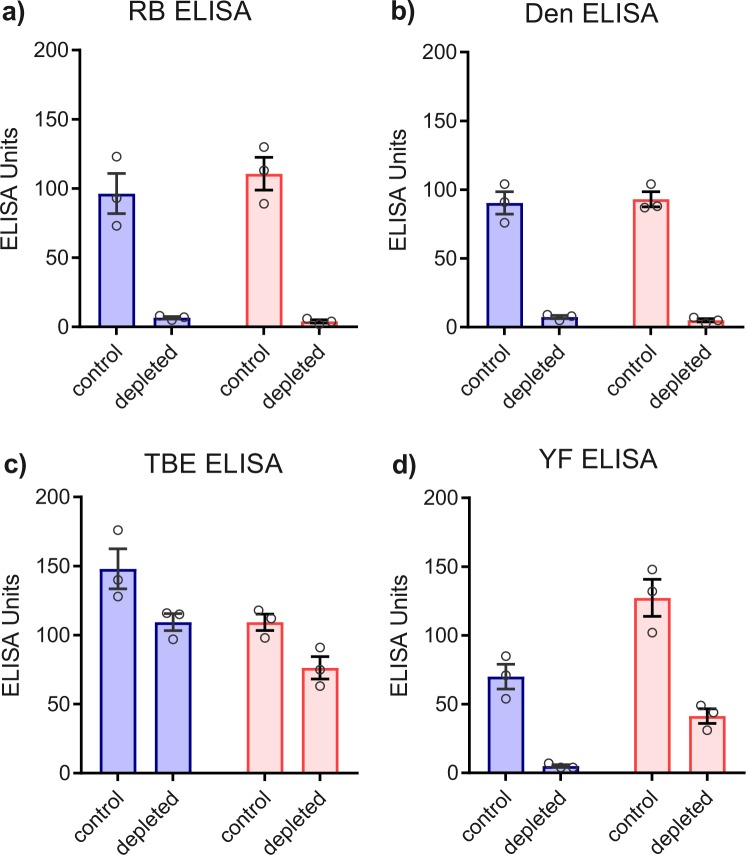
ELISA of TBE post-vaccination plasma pools at time point 28 weeks before (control) and after depletion of cross-reactive antibodies with RB sE. Blue columns (mean values): Plasma pool of flavivirus-naive group. Red columns (mean values): Plasma pool of YF pre-vaccinated group. Open circles show results of independent experiments. **a** RB ELISA. **b** Den ELISA. **c** TBE ELISA. **d** YF ELISA. Error bars represent the SEM calculated from three independent experiments

**Fig. 5:**
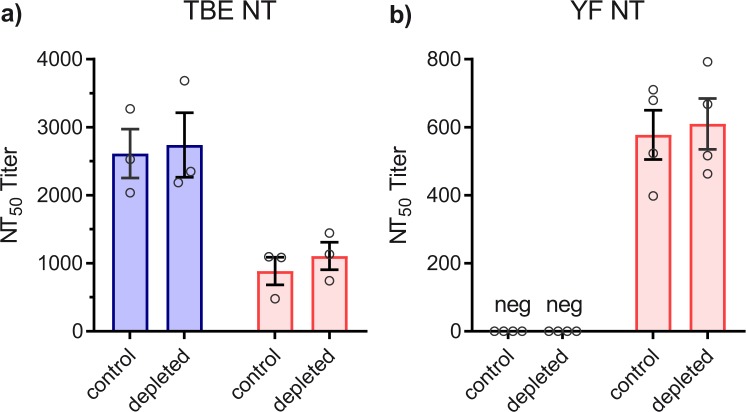
NTs of TBE post-vaccination plasma pools at time point 28 weeks before (control) and after depletion of cross-reactive antibodies with RB sE. Blue columns (mean values): Plasma pool of flavivirus-naive group. Red columns (mean values): Plasma pool of YF pre-vaccinated group. Open circles show results of independent experiments. **a** TBE NT. **b** YF NT. Error bars represent the SEM calculated from at least three independent experiments. No statistically significant difference was observed before and after depletion (measured by *t*-test)

To quantify the contributions of broadly flavivirus cross-reactive antibodies to ELISA and NT, we depleted the plasma pools obtained from both groups after the 3^rd^ vaccination with the E protein from RB virus and performed comparative analyses of pre- and post-depletion plasma pools in ELISA with RB, Den 1, TBE, and YF E proteins (Fig. 4a–d), as well as in NT with TBE and YF viruses (Fig. 5a, b). In the naive group, RB E-depletion resulted in complete loss of RB, Den and YF E reactivity, consistent with a common cross-reactive antigenic site in the E proteins of all three viruses (Fig. 4a, b, d). As shown in TBE ELISA (Fig. 4c), the contribution of such cross-reactive antibodies to the total TBE E response was relatively low, making up approximately 25% of ELISA-reactive antibodies. The YF pre-vaccinated pool revealed similar patterns as the naive pool except for the fact that depletion did not completely remove YF-reactive antibodies, consistent with the presence of both type-specific and cross-reactive antibodies after YF vaccination. RB depletion had no significant effect on the neutralization of TBE (Fig. 5a) and YF viruses (Fig. 5b), indicating that broadly flavivirus cross-reactive antibodies do not play an important role in the neutralization of these viruses.

### Analysis of single plasma samples

**Fig. 6:**
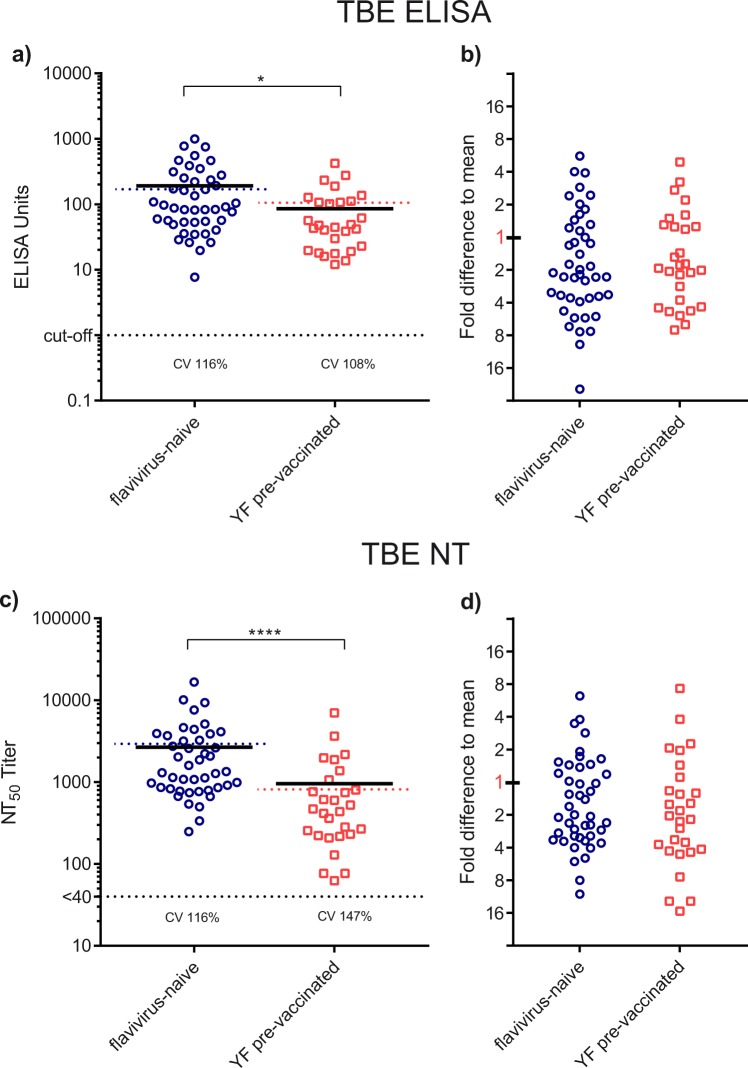
TBE ELISA and NT of individual TBE post-vaccination plasma samples at time point 28 weeks. Blue circles: Plasma samples from flavivirus-naive individuals. Red squares: Plasma samples form YF pre-vaccinated individuals. The values of the symbols represent the means of three to four independent experiments. Solid lines: Means of individual values. Dotted lines: Results of plasma pools. **a** TBE ELISA. **b** Fold differences of individual ELISA values to the means of all ELISA titers. **c** TBE NT. **d** Fold differences of individual NT titers to the means of all NT titers. Asterisks in **a** and **c** indicate significant differences between the two groups (measured by *t*-test): *P < 0.05; ****P < 0.0001

To obtain information about possible individual divergence from the results obtained with the plasma pools we performed TBE ELISAs and NTs with all plasma samples of the two groups (*n* = 44 and 28, respectively; Table 1) collected after the 3^rd^ vaccination (time point 28 weeks). The results are presented as individual ELISA values and NT titers (Fig. 6a, c) as well as the fold difference of each individual value to the mean of all values (Fig. 6b, d). The arithmetic means of single plasma samples (solid lines in Fig. 6a, c) matched the data obtained with the plasma pools (dotted lines in Fig. 6a, c), both in ELISA and NT. Although all individual samples were TBE NT positive, the impairment of the antibody response in the YF pre-vaccinated group, as observed with the plasma pool data, was confirmed in this analysis not only in NT (mean titers of 2683 vs.958) (Fig. 6c), but reached statistical significance also in ELISA (mean units of 193 vs. 86) (Fig. 6a). Despite this overall congruence of data, substantial deviations from the means were observed with individual plasma samples, and the difference between two individual samples could be as high as 130-fold (Fig. 6b, d).

**Fig. 7:**
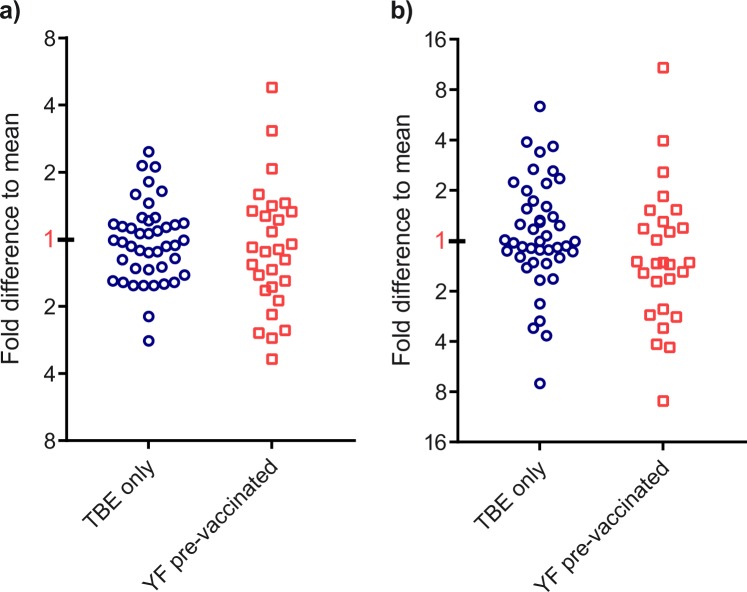
Fold differences of TBE ELISA **a** and TBE NT **b** to RB ELISA titers of individual TBE post-vaccination sera at time point 28 weeks. Blue circles: Plasma samples from flavivirus-naive individuals. Red squares: Plasma samples form YF pre-vaccinated individuals. The values of the symbols represent the means of three independent experiments. Ratios of TBE ELISA and NT titers to RB titers were calculated and expressed as the fold difference to the respective mean of the two groups

Individual differences in immune responses may not only affect the overall antibody titers but also shape antibody fine-specificities and influence the contribution of broadly cross-reactive antibodies to the total antibody response. To obtain information of such possible variations, we also determined the cross-reactive ELISA values using RB E and calculated ratios relative to the respective TBE ELISA values as well as to NT for each individual sample. The results, shown in Fig. 7 as fold difference to the means of all samples, revealed substantial deviations from the mean values, which were more pronounced in the YF pre-vaccinated group.

**Fig. 8:**
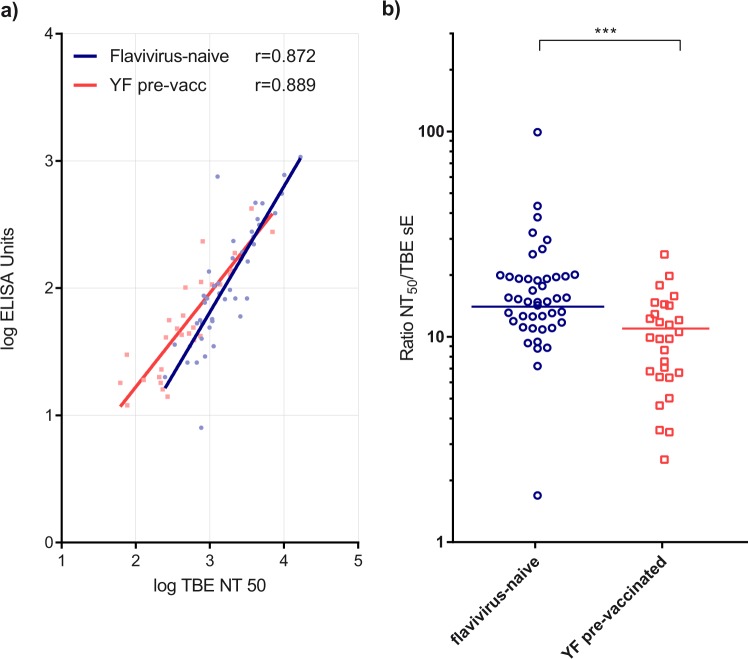
Correlation of ELISA and NT of TBE post-vaccination plasma samples at time point 28 weeks. Blue lines and circles: Plasma samples from flavivirus-naive individuals. Red lines and squares: Plasma samples from YF pre-vaccinated individuals. The values of the symbols represent the means of three to four independent experiments. **a** Linear regression of TBE ELISA and TBE NT_50_ titers. Spearman correlation coefficients (r) are indicated for both groups. **b** Ratios of TBE NT_50_ titers and TBE ELISA titers of individual plasma samples of both groups. Solid line: means of all single sera. Asterisks in **b** indicate significant differences between the two groups (measured by *t*-test): ***P < 0.001

Deviations in the proportions of antibody populations to different antigenic sites can potentially affect the specific neutralizing activity (ratio of NT to ELISA) of plasma samples. We therefore determined the correlation between TBE ELISA and NT values for all samples in both groups. As demonstrated in Fig. 8a, we found excellent correlations in both groups, confirming the usefulness of quantitative ELISA data as surrogates of TBE vaccine-induced immunity. However, two observations deserve attention in this context. i. The slope of the regression line is lower in the YF pre-vaccinated group, consistent with an overall higher proportion of cross-reactive and non-neutralizing antibodies in the plasma samples from this groups (Fig. 8a). This interpretation is supported by a significantly lower ratio of NT titers to ELISA values in the YF pre-vaccinated group than in the naive group (Fig. 8b). ii. In both groups, there are several outliers from the regression lines (Fig. 8a) and NT-to-ELISA ratios (Fig. 8b), revealing individuals with exceptionally high or low specific neutralizing activities.

### Possible other factors influencing antibody responses to TBE vaccination

Immune responses can be modulated by a variety of factors, including age and gender.^26^ We therefore stratified the TBE NT titers of each individual after the 3^rd^ vaccination (time point 28 weeks) with respect to these parameters, and the data are shown in Supplementary Fig. 1. None of these factors had an influence on the results obtained, and most importantly, all subgroup analyses confirmed the observed impairment of neutralizing antibody responses in the YF pre-vaccinated group. In addition to these factors, the time span between YF and TBE vaccination and/or the YF NT titer at the time point of TBE vaccination could also influence the degree of impairment observed in the YF pre-vaccinated group. However, as shown in Supplementary Fig. 2, these two parameters did not correlate with the extent of the TBE virus neutralizing antibody response.

## Discussion

Our analyses show that the immune response to TBE vaccination can be substantially altered by pre-existing YF vaccine-induced immunity. Most importantly, we found that the TBE-specific neutralizing antibody response was impaired compared to a flavivirus-naive control group, whereas non-neutralizing antibodies to broadly flavivirus cross-reactive epitopes were boosted and higher in the initial phase of vaccination. Nevertheless, all individuals had TBE NT titers > 40 after the recommended vaccination course, irrespective of previous YF-vaccination. Since positive serum neutralization values are considered to be protective,^19,27^ the data suggest that the current vaccination schedule is sufficient also in YF pre-vaccinated individuals, at least if the interval between YF and TBE vaccination is relatively long, as in our group. However, quantitative differences as found in our analyses may have implications for long-term protection and highlight the need for a comparative assessment of antibody persistence and vaccine efficacy in both flavivirus-naive and pre-immune individuals. Such assessments are especially important for the recommended schedules of revaccination intervals, which are currently five years (three years in the elderly) and do not take into account pre-existing flavivirus immunity.^19^

The effects of immunological memory on the antibody response may vary with different combinations of pre-existing immunity and heterologous flavivirus vaccination. For instance, the neutralizing antibody response to JE vaccination was found to be superior in TBE pre-vaccinated than in naive individuals, but only after the first and not after the second vaccination.^28^ A trend towards higher NT titers in individuals with pre-existing flavivirus immunity (albeit non-significant) was also found in a recent Zika vaccination study.^29^ These variations and discrepancies certainly deserve further investigation and may be related to the specific antigenic relationship between pairs of flaviviruses and/or the characteristics of the vaccines as well as the viruses and assay systems used in these comparisons.

The increase of broadly flavivirus cross-reactive antibodies in the YF pre-vaccinated group can be explained by the existence of memory B cells targeting conserved epitopes that are rapidly stimulated to become antibody-secreting plasma cells upon TBE vaccination. A similar boosting of such antibodies, concomitant with a broadening of the antibody response, was also observed in a TBE vaccination study with YF pre-immune individuals,^30^ in YF vaccinated persons with pre-existing immunity to heterologous flaviviruses^17^ as well as in studies of sequential flavivirus infections.^31,32^ The primary target of broadly flavivirus cross-reactive antibodies is the highly conserved fusion loop (FL) at the tip of domain II in E (Fig. 1a, b). Because of its interdigitation with a hydrophobic pocket provided by the second subunit in the E dimer, the accessibility of this sequence element can be restricted at the surface of mature infectious virions, and this epitope has therefore been referred to as ‘cryptic’.^6,33^ Since neutralization of neither TBE nor YF virus was impaired after removal of the cross-reactive antibody population from post-vaccination plasma samples (Fig. 5), it can be assumed that the FL is relatively inaccessible in both viruses. This finding is an important distinction to dengue viruses, which were shown to be neutralized by broadly cross-reactive monoclonal and polyclonal antibodies,^6,34–36^ including those induced by TBE vaccination.^30^ The accessibility of this broadly cross-reactive epitope thus appears to vary strongly among flaviviruses, depending on the stability and maturation state of the virus as well as the extent of ‘virus breathing’, which can lead to a transient exposure of the FL and thus facilitate interactions with antibodies.^6,20^

The impaired neutralizing activity of plasma samples from the YF pre-immune group (evidenced both by comparatively lower TBE NT titers and by lower NT/ELISA ratios; Fig. 8) may be the result of several mechanisms that act in concert during immune responses in the absence or presence of cross-reactive immunity. A key discriminatory element in the YF pre-vaccinated group is the potential of forming immune complexes between pre-existing cross-reactive antibodies and the TBE vaccine antigen. Immune complexes can interact with Fcγ receptors on immune cells and trigger processes that may influence processing and antigen presentation to T cells, trafficking to germinal centers, affinity maturation and selection of B cells,^13^ all of which can lead to competition between memory and naive responses. Further mechanisms contributing to an impaired neutralizing antibody response by cross-reactive antibodies may be related to ‘carrier-induced epitopic suppression’^37^ and/or the shielding of epitopes adjacent to the FL that could otherwise give rise to potently neutralizing antibodies.

In addition to feedback mechanisms mediated by pre-existing antibodies, the impaired neutralizing antibody response observed in YF pre-immune TBE vaccinees may also be related to differences in the stimulation of CD4 helper T cells. While CD4 follicular helper cells (Tfh) are essential for an effective germinal center (GC) reaction, needed for the effective priming of novel antibody specificities, another subset (CD4 follicular regulatory cells - Tfr) has been identified that controls the GC response and can potently inhibit B cell responses.^38^The outcome of de novo antibody production in the course of sequential flavivirus vaccination may thus be negatively affected by the re-stimulation of CD4 memory cells that impair B cell responses by producing inhibitory cytokines or mediating other immunosuppressive mechanisms.^38^ It can be expected that such CD4 memory effects would be influenced by a number of factors, including the type of vaccine (replicating or non-replicating) used in primary and secondary vaccination as well as the extent of shared T cell epitopes among different vaccine antigens.

Of concern in the context of defining correlates of protection^39^ is the lower ratio of TBE NT titers to ELISA values observed in YF pre-vaccinated individuals. Although an excellent correlation between these parameters was found in both groups (Fig. 8a), the slope of the regression line was less steep in the YF pre-vaccinated group than in the naive group, consistent with a higher proportion of cross-reactive antibodies reactive in ELISA relative to TBE virus-specific neutralizing antibodies. Since broadly cross-reactive antibodies did not contribute to TBE virus neutralization (Fig. 5), their disproportionate presence can introduce a bias in using ELISA data for measuring vaccine responses. Such a bias would be even more pronounced with hemagglutination-inhibition (HI) tests, because flavivirus hemagglutination is caused by the exposure of the FL and its interaction with red blood cells under the acidic pH conditions of the assay.^6,40^ Broadly cross-reactive, FL-specific antibodies are therefore most effective inhibitors of flavivirus hemagglutination, explaining the pronounced broadening of HI reactivities observed previously in studies of sequential flavivirus immunization or infection.^30,41^

Overall, in both of our groups, broadly cross-reactive antibodies did not dominate the antibody response after the last vaccination, making up only 20 to 30 % of the total TBE E-reactive antibodies measured in ELISA (Fig. 4). These data are consistent with previous analyses of cross-reactive antibodies after TBE and YF vaccination^42,43^ and a recent study showing that TBE vaccine-induced antibodies mediate only low antibody-dependent enhancement of Zika virus infection in vitro and negligible enhancement in vivo.^44^ Nevertheless, the boosting of broadly cross-reactive antibodies through sequential flavivirus vaccinations deserves attention and may be of concern because of the role of these antibodies in phenomena of infection and disease enhancement, especially in dengue virus infections.^6,8^ In specific instances, however, infection enhancement by cross-reactive antibodies might have beneficial effects, as shown by an increased immunogenicity of YF vaccine in individuals with a pre-existing Japanese encephalitis immunity due to prolonged viremia.^45^

Of note, higher values of broadly cross-reactive antibodies in the YF pre-vaccinated group were observed only in the initial phase of immunization (after the first and second vaccination), but not after the booster at 24 weeks, when these antibodies reached similar levels in the flavivirus-naive group (Fig. 3). One possible explanation would be that the amounts of cross-reactive antibodies present in the YF pre-vaccinated group were already sufficiently high to mask the epitope and thus to prevent a further increased boost of cross-reactive antibodies relative to the flavivirus-naive control group. Such a mechanism of ‘epitope masking’ has been proposed (based on modeling studies) in the context of limited boosting of antibodies to conserved epitopes on the stem of the influenza hemagglutinin.^46^

An especially interesting aspect of our analyses is the strong variability of individual responses observed in both groups, resulting in substantial deviations from the mean ratios of i. broadly cross-reactive to TBE virus-specific antibody titers (Fig. 7) and ii. TBE NT titers to TBE ELISA values (Fig. 8). Both parameters reflect variations of the epitope specificities of antibody populations present in individual plasma samples. Since all individuals had been vaccinated with the same vaccine and therefore encountered precisely the same immunogen, this variation may be the result of stochastic processes that operate at different stages of the immune response, including competition of B cells in germinal centers, selection of certain B cells to become long-lived plasma cells in the bone marrow and feedback from antibodies^47^ as well as antagonistic effects caused by CD4 Tfh and Tfr memory T cells.^38^

In addition, human immune responses can be influenced by other factors, including but not restricted to age and sex.^26^ Breaking down our groups into individuals older and younger than 50 years, and women and men, did not yield significant differences of TBE NT titers related to these parameters. Importantly, in all subgroup-comparisons, a significantly lower TBE NT titer was found in those pre-vaccinated against YF (Supplementary Fig. 2), corroborating the main conclusion drawn from overall group analyses. There was also neither positive nor negative correlation between time elapsed since YF vaccination or the titers of YF neutralizing antibodies at the time of initial TBE vaccination and post-vaccination TBE NT titers (Supplementary Fig. 2). We therefore can conclude that pre-existing YF cross-reactive immunity in general was the key factor leading to an impaired neutralizing antibody response to TBE vaccination.

In conclusion, our work points to a potential negative influence of pre-existing cross-reactive immunity on the efficacy of flavivirus vaccination and draws attention to important individual differences in the fine-specificities of antibody responses that affect virus neutralization. Based on the data shown, an increased awareness of the possible impact of pre-existing flavivirus immunity in the assessment of flavivirus vaccines appears to be warranted. Further studies will be required to evaluate whether the phenomena observed with TBE vaccination and YF immunity also apply to other combinations of flavivirus vaccines and/or infections and to what extent the time interval between administration of antigenically related vaccines is crucial for protective immunity.

## Methods

### Plasma samples from TBE-vaccinated individuals

The current work is a retrospective and anonymized analysis of samples from individuals that had participated under informed consent in a TBE vaccination study (FSME-Immun® CC) originally conducted in 2007/2008 at the Institute of Social and Preventive Medicine (now Epidemiology, Biostatistics and Prevention Institute), University of Zurich and at the Division of Infectious Diseases, University Hospital of Zurich, Switzerland. For the present analysis, samples from 72 out of 159 individuals were selected according to the following criteria: Documented YF vaccination (*n* = 28) OR no YF vaccination AND no travel history to YF endemic areas (*n* = 44); no record or evidence of other flavivirus infections or vaccinations; no history of previous tick bites; fully negative TBE-specific IgG measured by ELISA at baseline.

All individuals had received three doses of an inactivated TBE vaccine over the course of six months, according to the schedule displayed in Fig. 1c. Blood was taken at the time points indicated in Fig. 1c and samples were sent to the Center for Virology, Medical University of Vienna, Austria, for anonymized analyses. All plasma samples were heat-inactivated for 30 min at 56 °C prior to serological testing. Pools were prepared from equal aliquots of inactivated plasma samples.

### Production of recombinant proteins

The sE proteins of YF virus strain 17D (GenBank accession number X03700, aa 1–397), TBE virus strain Neudoerfl (GenBank accession number U27495, aa 1–400), RB virus strain RiMAR (GenBank accession number AF144692, aa 1-394) and Den 1 virus strain FGA/89 (GenBank accession number AF226687, aa 1-399), each containing a C-terminal strep-tag, were produced in Drosophila Schneider 2 (S2; Invitrogen) cells.^43^ For this purpose, we used an expression vector (pT389, kindly provided by Felix Rey, Institut Pasteur, France) that encodes the export signal sequence Bip, an enterokinase cleavage site and a double strep-tag. S2 cells were stably transfected with the expression vector, using blasticidin for selection. Protein expression was induced by the addition of CuSO_4_ and supernatants were harvested 7-10 days after induction. Recombinant proteins were purified via affinity chromatography with Strep-Tactin columns (IBA Lifesciences) according to the manufacturer’s instructions.

### Quantification of flavivirus E-protein specific IgG antibodies by ELISA

Purified recombinant E proteins of TBE, YF, Den 1 or RB viruses carrying a strep-tag were added to Strep-Tactin coated microtiter plates (IBA Lifesciences) at a concentration of 0.5 µg/ml and incubated for 1 h at 37 °C. After blocking the plates with 1% bovine serum albumin (BSA) in phosphate-buffered saline (PBS) pH 7.4 for 30 min at 37 °C, threefold serial dilutions of plasma samples (from 1∶100 to 1:300,000) were added and incubated for 1 h at 37 °C. Using these dilutions, endpoints were reached in all instances. Bound antibodies were detected with a goat anti-human IgG conjugated to horseradish peroxidase (Thermo Fisher Scientific, catalog # 31412, dilution 1:5000). Specific antibodies were quantified using a flavivirus IgG-positive human serum as an internal standard, arbitrarily defined to contain 1000 IgG Units. The serum was derived from a vaccinated individual and had a TBE NT_50_ titer of 7245, and the ratio of the two parameters was within the range observed with the samples analyzed in our study. Since the standard was included on each plate of the assays, this approach allowed controlling plate-to-plate variation and thus further improved reproducibility compared to titer determinations. Curve fitting was performed with Prism version 7 (GraphPad Software Inc.). In each test, the cut-off was defined as the means plus three standard deviations of values obtained with a panel of 8 flavivirus-negative human sera.^48^

### Reporter virus production

TBE reporter virus particles (RVPs) were produced in human embryonic kidney cells (HEK293T/17, ATCC) similar to the production of WN or Den RVPs.^49,50^ Briefly, cells were transfected with two plasmids, a WN virus sub-genomic replicon (kindly provided by Ted Pierson; Viral Pathogenesis Section, National Institute of Allergy and Infectious Diseases, National Institutes of Health, Bethesda, MD, USA), carrying the Renilla luciferase gene, and an expression plasmid carrying the structural genes of TBE virus. This plasmid was based on the pRLSV40 vector (Promega) in which the Renilla luciferase gene was replaced by the coding sequence for the structural proteins capsid (C), premembrane (prM) and envelope (E) from TBE virus strain Neudoerfl (GenBank accession no. U27495). The two plasmids were transfected at a ratio of 1:3 with Lipofectamin LTX Plus (Invitrogen) according to the manufacturer’s instruction. Four hours after transfection, cells were washed and further incubated for 48 h in low-glucose Dulbecco’s Modified Eagle Medium (DMEM) buffered with 25 mM HEPES (Gibco). The cell culture supernatant containing TBE RVPs was harvested, clarified through a 0.22 µm filter (MF-Millipore) and stored at −80 °C until used.

### Neutralization test using TBE reporter viruses

Reporter virus neutralization tests were carried out in 96-well flat-bottom plates (Corning) using HEK cells (HEK293T/17, ATCC). TBE RVPs were diluted in DMEM (2.5% FCS, 1% glutamine, 0.5% neomycin; Gibco) to a concentration corresponding to a luciferase intensity of 1000 light units. Triplicates of 2-fold dilution series of individual or pooled plasma samples (starting at 1:20) were incubated with an equal volume of RVPs for 1 hour at 37 °C (the lowest plasma dilution tested was therefore 1:40).Then 4 × 10^4^ cells were added to each well and incubation was continued for 48 h at 37 °C. Luciferase activity was measured using the Renilla-Glo Luciferase assay system (Promega) according to manufacturer’s instruction in a 1420 Luminescence Counter (Perkin Elmer). Neutralization titers were determined at a cut-off of 50% reduction of the luminescence signal (NT_50_ titers), using a four-parameter logistic regression for curve fitting (GraphPad Prism 7; GraphPad Software Inc.).

### YF virus neutralization test

YF virus-specific neutralizing antibodies were determined in baby hamster kidney cells (BHK-21, ATCC), essentially as described by.^43^ In brief, triplicates of three-fold dilution series (starting at a dilution of 1∶50) of individual or pooled plasma samples were mixed with 20–40 TCID_50_ of YF 17D vaccine virus in minimum essential medium (MEM) (2.5% FCS, 1% glutamine, 0.5% neomycin) and incubated for 1 h at 37 °C prior to addition of cells and further incubation for 72 h at 37 °C. The cells were then fixed with 4% paraformaldehyde and virus-infected cells were detected using a YF virus-specific mouse monoclonal antibody (1 µg/ml 2D12; affinity-purified from ATCC CRL-1689 hybridoma cells) in combination with an Alexa Fluor 488 labeled rabbit anti-mouse IgG (Invitrogen, catalog # A11059, dilution 1:500). Fluorescence was measured in a Synergy HTX plate reader (BioTek). Neutralization titers were determined at a cut-off of 50% reduction of the fluorescence signal (NT_50_ titers), using a four-parameter logistic regression for curve fitting (GraphPad Prism 7; GraphPad Software Inc.).

### Antibody depletion

Depletion of cross-reactive antibodies from plasma samples was performed with strep-tagged sE from the distantly related RB virus, coupled to Strep-Tactin magnetic beads (IBA Lifesciences).^42,51^ The beads were incubated in PBS buffer containing 0.1 % BSA with a 1∶5 dilution of plasma for 1 h at 37 °C. Beads were removed by magnetic force and the supernatant was collected. This depletion procedure was performed three times. Beads without coupled sE were used as a control for non-specific binding.

### Statistical analyses

Statistical analyses were performed with GraphPad Prism 7 (GraphPad Software Inc.). Logarithmic transformation of the data was carried out to obtain approximate normal distribution of antibody concentrations and NT titers. Two-tailed *t*-tests were applied to the transformed data for significance testing, and correlation coefficients were determined with the Spearman correlation test. *P* values < 0.05 were regarded as statistically significant.

### Reporting Summary

Further information on research design is available in the Nature Research Reporting Summary linked to this article.

## Supplementary information

Reporting Summary

Supplemental Figures

## Data Availability

All datasets used and/or analyzed in the current study are available from the corresponding author upon reasonable request.

## Supplementary information

**Supplementary information** accompanies the paper on the *npj Vaccines* website (10.1038/s41541-019-0133-5).
