# Supplementary material for: Pre-existing yellow fever immunity impairs and modulates the antibody response to tick-borne encephalitis vaccination
Source: NPJ Vaccines. 2019 Sep 6;4:38. doi: 10.1038/s41541-019-0133-5 (PMC6731309; doi:10.1038/s41541-019-0133-5)
Supplement: Supplementary file 2 — Supplemental Figures [file 41541_2019_133_MOESM2_ESM.pdf]

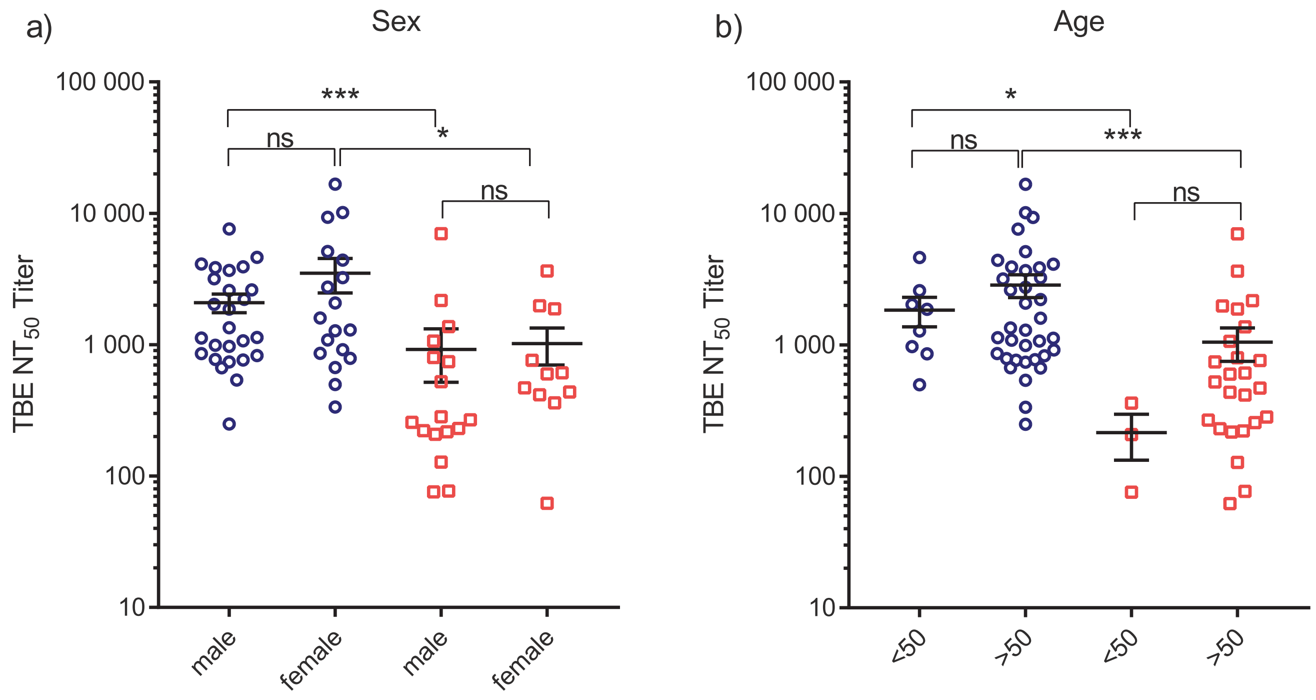


**Supplemental Figure 1: TBE NT titers at time point 28 weeks of individual plasma samples of both groups stratified according to sex (a) and age (b).** Blue circles: Plasma samples from flavivirus-naïve individuals. Red squares: Plasma samples from YF pre-vaccinated individuals. The values of the symbols represent the means of three to four independent experiments. Black lines indicate mean values and error bars represent the standard error of the means (SEM). Asterisks indicate significant differences (measured by t-tests): *, P<0.05; ***, P<0.001; ns, not significant.

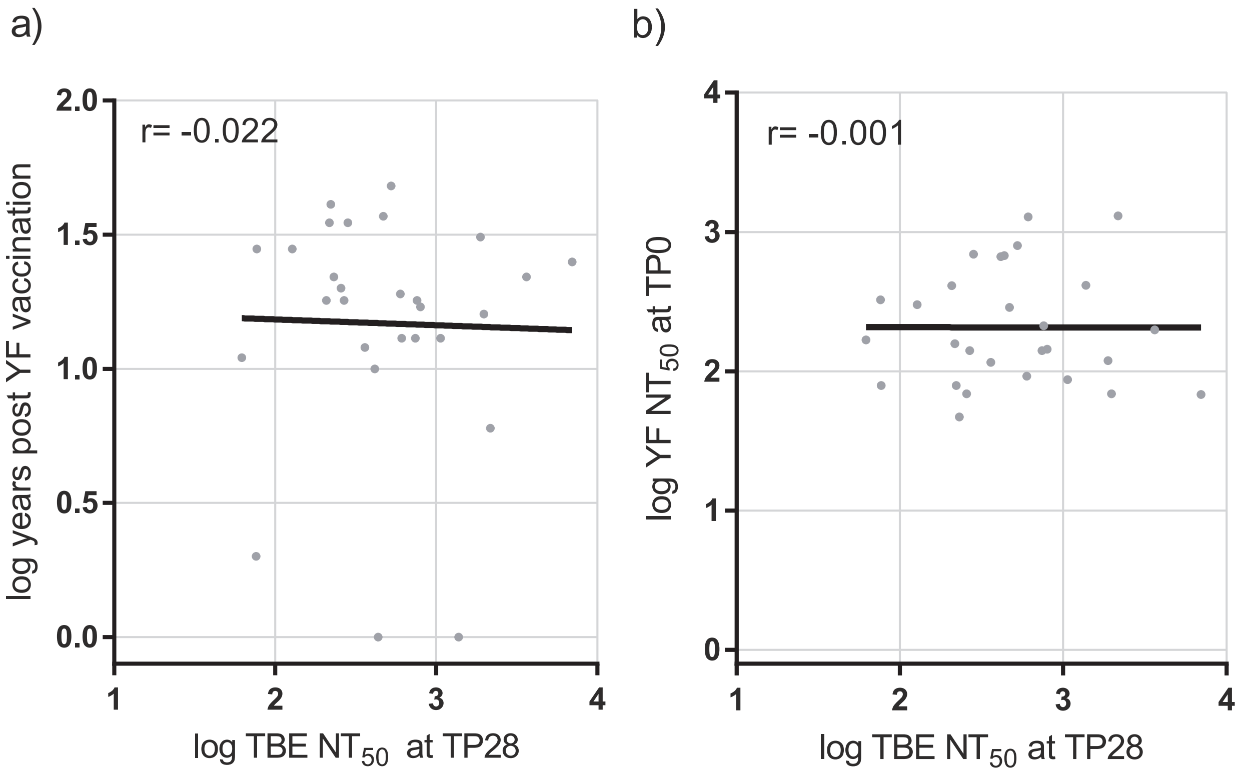


**Supplemental Figure 2: Correlation of TBE NT titers at time point 28 weeks and (a) time elapsed since YF vaccination and (b) YF NT titers at the time of first TBE vaccination in the YF pre-vaccinated group.**

Linear regression lines are shown and Spearman correlation coefficients (r) are indicated in both panels.
